# Different Calcium and Src Family Kinase Signaling in Mac-1 Dependent Phagocytosis and Extracellular Vesicle Generation

**DOI:** 10.3389/fimmu.2019.02942

**Published:** 2019-12-17

**Authors:** Ákos M. Lőrincz, Viktória Szeifert, Balázs Bartos, Dávid Szombath, Attila Mócsai, Erzsébet Ligeti

**Affiliations:** Department of Physiology, Semmelweis University, Budapest, Hungary

**Keywords:** extracellular vesicles, phagocytosis, neutrophils, signaling, antibacterial effect, calcium, Src family kinase, complement receptor

## Abstract

Encountering opsonized particles by neutrophils results in phagocytosis of the particle and generation of extracellular vesicles with antibacterial property (aEV). The aim of the present study is to compare the involvement of different receptors and receptor-proximal signaling pathways in these two parallel processes. Investigating human neutrophils from peripheral blood, we show that complement receptors are decisive for both processes whereas immunoglobulin binding Fc receptors (FcR) only participate moderately in phagocytosis and pattern recognition receptors induce mild EV production but only minimal phagocytosis. Studying bone marrow derived neutrophils of genetically modified animals we verify that the involved complement receptor is CR3, also known as the β_2_ integrin Mac-1. We show that genetic deletion of the adaptor molecules FcRγ chain or DAP12 does not influence either process, suggesting potential redundant function. Combined absence of the Src family kinases Hck, Fgr, and Lyn drastically impairs phagocytosis but does not influence aEV production. In contrast, deletion of PLCγ2 has no influence on phagocytosis, but reduces aEV formation. In accord with the essential role of PLCγ2, aEV biogenesis both from murine and from human neutrophils is dependent on presence of extracellular calcium. Absence of external calcium prevented the generation of antibacterial EVs, whereas the spontaneous EV formation was not influenced. We thus show that phagocytosis and biogenesis of antibacterial EVs are independent processes and proceed on different signaling pathways although the same receptor plays the critical role in both. Our data reveal the possibility in neutrophilic granulocytes to modulate aEV production without disturbing the phagocytic process.

## Introduction

Phagocytosis by neutrophilic granulocytes is significantly promoted by opsonization. The most effective opsonins are immunoglobulins reacting with different Fc receptors (FcR) and complement fragments recognized by complement receptors (CR). The most abundant CR on the surface of neutrophils is CR3, also known as macrophage antigen 1 (Mac-1), a β_2_ integrin (1). In addition to phagocytosis, Mac-1 is involved in adhesion and spreading of the cells as well as in adhesion-dependent superoxide production and degranulation (2–4). Recently we found that Mac-1 also plays a critical role in production of extracellular vesicles with antibacterial capacity (5).

Generation of extracellular vesicles (EVs) is a common property of different cell types from the simplest prokaryotes up to the most differentiated eukaryotic cells (6, 7). Investigation of the physiological and pathological roles of EVs has skyrocketed in the last decade and by now EVs are considered as important elements of intercellular communication. Some of the established functions of different EV types such as antigen presentation (8), anti-inflammatory (9), or antimicrobial effects (10) represent protective mechanisms for the host organism. Others, such as transfer of oncogenic receptors (11) or dissemination of antibiotic resistance (12) are more frightening. Hence, medical therapy may require - depending on the exact condition - enhancement or reduction of EV production. For the time being such interventions are hampered by the lack of knowledge on the molecular mechanisms of biogenesis.

Diversity of EVs has been widely documented (13, 14). There are clear examples indicating that properties of EVs released even from the same cell vary depending on environmental factors (15–17). Investigating EV production from neutrophilic granulocytes, we have previously characterized three different types of medium-sized EVs (also called microvesicles or ectosomes): those produced from freshly isolated cells spontaneously (sEV), or upon activation with opsonized particles (aEV) and EVs produced during apoptosis of the cells (apoEV). We found differences in the number, protein content and protein composition of these EV populations (18). The most striking difference was in their functional property, as aEVs impaired the growth of bacteria in a concentration-dependent way whereas the other two types had absolutely no antibacterial effect (5). However, the cellular processes governing the production of these divergent EV populations are poorly explored.

Searching for the molecular mechanism of generation of antibacterial EVs, we identified Mac-1 as the key cell surface receptor triggering aEV production. The aim of the current study was to investigate the relationship of the two Mac-1 dependent processes and to characterize the initial steps of the signaling pathway leading from receptor activation eventually to the release of aEVs or phagocytosis. Using neutrophils isolated from human peripheral blood or from genetically modified mice, we show that the two processes are independent and proceed on clearly distinguishable signaling pathways.

## Materials and Methods

### Materials

HBSS with or without calcium and magnesium and glucose was from GE Healthcare Life Sciences (South Logan, UT, USA), Zymosan A from Sigma Aldrich (St. Louis, MO, USA), Ficoll-Paque and Percoll from GE Healthcare Bio-Sciences AB (Uppsala, Sweden), HEPES (pH 7.4) from Sigma. All other used reagents were of research grade. GFP-expressing and chloramphenicol resistant *S. aureus* (USA300) was a kind gift of Professor William Nauseef (University of Iowa).

### Preparation of Human PMN and EV

Venous blood samples were drawn from healthy adult volunteers according to procedures approved by the National Ethical Committee (ETT-TUKEB No. BPR/021/01563-2/2015). Neutrophils were obtained by dextran sedimentation followed by a 62.5% (v/v) Ficoll gradient centrifugation (700*g*, 40 min, 22°C) as previously described (18). The preparations contained more than 95% PMN and <0.5% eosinophils. PMNs (typically 10^7^ cell in 1 mL HBSS) were incubated with or without activating agent for 30 min at 37°C in a linear shaker (80 rpm/min). After activation, cells were sedimented (500*g*, Hermle Z216MK 45° fixed angle rotor, 5 min, 4°C). Upper 500 μL of the supernatant was filtered through a 5 μm pore sterile filter (Sterile Millex Filter Unit, Millipore, Billerica, MA, USA). The filtered fraction was sedimented (15700 *g*, Hermle Z216MK 45° fixed angle rotor, 5 min, 4°C), and the pellet was carefully resuspended in the original incubation volume. Protein concentration of EV was determined by the Bradford protein assay using BSA as standard (5).

### Transgenic Mice

Triple Src-family kinase (SFK) knock-out mice in which all three Src-family kinase isoforms identified in neutrophils (Hck, Fgr, and Lyn) were missing (19) were obtained from Clifford Lowell (University of California, San Francisco, CA). Complete CD18-deficient (Itgb2^tm2Bay/tm2Bay^, referred to as CD18^−/−^) mice (20) were obtained from A. Beaudet (Baylor College of Medicine, Houston, TX). CD11b-deficient (*Itgam*^tm1Myd/tm1Myd^, referred to as CD11b^−/−^) mice were purchased from The Jackson Laboratory (21). CD11a-deficient (*Itgal*^tm1Hogg/tm1Hogg^, referred to as CD11a^−/−^) mice were obtained from N. Hogg (Cancer Research UK, London, UK) (19). FcRγ-chain deficient (*Fcer1g*^tm1Rav/tm1Rav^, referred to as FcRγ^−/−^) mice were purchased from Taconic (22). Dap12-deficient (referred to as Dap12^−/−^) mice (22) were obtained from Lewis Lanier (University of California, San Francisco, CA). Heterozygous mice carrying a deleted allele of the PLCγ2-encoding gene (Plcg2^tm1Jni^, referred to as PLCγ2^−/+^) were obtained from James N. Ihle (St. Jude Children's Research Hospital, Memphis, TN, USA) (23) and have been backcrossed to the C57BL/6 genetic background for more than 10 generations. Because of the limited fertility of homozygous PLCγ2^−/−^ mice, the mutation was maintained in heterozygous form as described (24). Syk^+/−^ mice (25) were originally obtained from Victor Tybulewicz (National Institute of Medical Research, London, UK). Syk^−/−^ bone marrow chimeras carrying a Syk-deficient hematopoietic system were generated by transplanting Syk^−/−^ fetal liver cells into lethally irradiated wild-type recipients (26). All transgenic mice were backcrossed to the C57BL/6 genetic background for at least 6 generations. All transgenic mice were 11–20 weeks old. Age- and sex-matched C57Bl/6 animals were used as controls. Genotyping was performed by allele-specific PCR. WT control C57BL/6 mice were purchased from Charles River or the Hungarian National Institute of Oncology. Mice were kept in sterile, individually ventilated cages (Tecniplast, Buguggiate, Italy) in a conventional facility. All animal experiments were approved by the Animal Care Committee of the National Authority for Animal Health (Budapest, Hungary).

### Isolation of Murine PMN and EV

Murine neutrophils were isolated from the bone marrow of the femurs, humeri, and tibias of intact mice by hypotonic lysis followed by Percoll gradient centrifugation (62.5% v/v, 700*g*, 40 min, 22°C) using sterile and endotoxin-free reagents as previously described (19). Cells were kept at room temperature in Ca^2+^ and Mg^2+^-free medium until use (usually <30 min) and pre-warmed to 37°C before activation. Neutrophil assays were performed at 37°C in HBSS supplemented with 20 mM HEPES, pH 7.4. PMNs (10^7^ cell in 1 mL HBSS) were incubated with or without activating agent for 30 min at 37°C on a linear shaker (80 rpm/min). After incubation, PMNs were sedimented (1,000 *g*, Hermle Z216MK 45° fixed angle rotor, 5 min, 4°C) and the upper 800 μL of the supernatant was filtered through a 5 μm pore sterile filter. The filtered fraction was sedimented again (30,000 *g*, Beckmann JA-17 fixed 25° angle rotor, 30 min, 4°C). The sediment was resuspended in HBSS at the original volume and used immediately for further analysis according to previous observations (27).

### Opsonization

Zymosan (5 mg in 1 mL HBSS) was opsonized with 500 μL pooled human or murine serum for 30 min at 37°C. For complement-free opsonization zymosan (5 mg in 1 mL PBS) was opsonized with 500 μL human serum pretreated with 20 mM EDTA. After opsonization zymosan was centrifuged (5,000*g*, 5 min, 4°C, Hermle Z216MK 45° fixed angle rotor), and washed once in HBSS.

Bacteria (OD_600_ = 1.0 in 800 μL HBSS) were opsonized with 200 μL pooled human or murine serum or with EDTA pretreated human serum for 30 min at 37°C. After opsonization, bacteria were centrifuged (8,000 *g*, 5 min, 4°C, Hermle Z216MK 45° fixed angle rotor), and washed once in HBSS.

### EV Analysis and Quantification by Flow Cytometry

Human EVs were labeled with RPE conjugated monoclonal anti-CD11b (1 μg/mL, Tonbo Biosciences, USA, clone M1/70) (28), FITC conjugated anti-CD18 monoclonal Ab (1 μg/mL, Dako) or FITC conjugated annexinV (BD Biosciences) for 20 min at 37°C and then washed in HBSS. Murine EVs were labeled with RPE conjugated monoclonal anti-CD11b (1 μg/mL, Tonbo Biosciences, USA, clone M1/70) (29) or RPE conjugated monoclonal anti-CD18 (1 μg/mL, BD Biosciences, clone C71/16) (30) or PerCP-CY 5.5 conjugated monoclonal anti-Ly6g (1 μg/mL, BD Biosciences, clone 1A8) (31) or FITC conjugated AnnexinV (BD Biosciences) for 20 min at 37°C and then washed in HBSS. Isotype controls were from identical manufacturer, annexinV labeling was controlled in 20 mM EDTA containing medium.

For flow cytometric detection of EVs a Becton Dickinson FACSCalibur flow cytometer was used as described previously in Lorincz et al. (27). Briefly pure HBSS medium was used for setting the threshold to eliminate instrument noise then fluorescent beads (3.8 μm SPHERO Rainbow Alignment Particles from Spherotech Inc., USA) were detected to set the upper size limit of EV detection range. After the measurement of an EV preparation the number of isotype control events and the 0.1% TritonX-100 detergent non-sensitive events were subtracted to calculate the true EV number. To avoid swarm detection, the flow rate was held below 1,000 events/s (3,750 events/μL) during measurements. Samples were re-measured after a 2-fold dilution to control linearity of measurements. Linearity was controlled in a broader range previously (32). FC data were analyzed with Flowing 2.5 Software (Turku Center for Biotechnology, Finland).

### Quantification of Phagocytosis

Neutrophils (10^6^ in 1 mL HBSS) were incubated with GFP expressing *S. aureus* (*USA300*, 10^7^ in 1 mL HBSS) for 30 min at 37°C in a linear shaker (80 rpm/min). For kinetic measurements samples were taken in every 10 min, for endpoint measurements phagocytosis was stopped after 20 min. Samples were diluted 5-fold in ice-cold PBS and analyzed by flow cytometry. To avoid coincidental co-detection of cells and bacteria and to control linearity of the measurement every sample was measured again after a 2-fold dilution. Percentage of phagocytosing PMNs was calculated by detecting GFP positive and negative cells.

**Figure 1:**
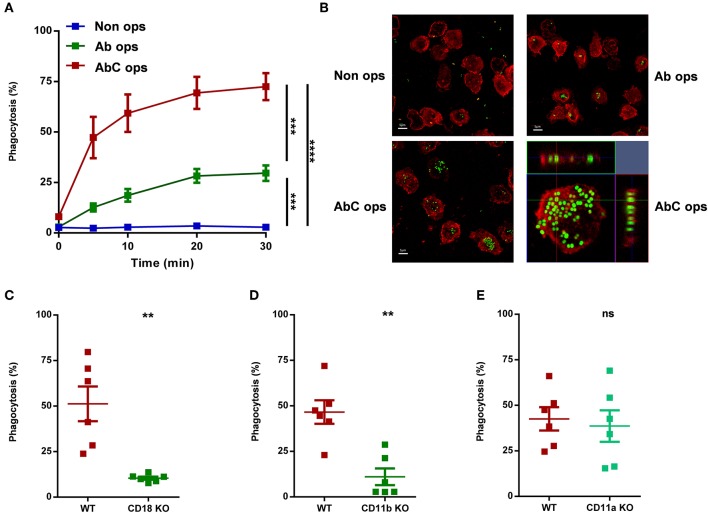
Participation of different receptors in phagocytosis of human **(A,B)** or murine **(C–E)** neutrophils. **(A)** Analysis of phagocytosis of non-opsonized, partially, or completely opsonized GFP expressing *S. aureus* by human PMN. Kinetics of phagocytosis ± SEM, *n* = 4. Data were compared after 30 min phagocytosis using RM-ANOVA coupled with Tukey's *post hoc* test. **(B)** Confocal microscopic images of human neutrophils after 20 min phagocytosis of non-opsonized (UL), partially (UR), and completely (LL) opsonized GFP expressing *S. aureus*; red color shows CD11b labeling. X-Y projections of engulfed bacteria with the respective side views (LR). Representative images out of 4 independent experiments. **(C–E)** Quantification of phagocytosis of WT vs. CD18 KO **(C)**, CD11b KO **(D)**, CD11a KO **(E)** murine PMN. Data were compared using Student's *t*-test; *n* = 6, 6, 6 ± SEM. **P < 0.01; ***P < 0.001; ****P < 0.0001.

To prove that cell associated bacteria are internalized (and not only bound by the surface), we carried out confocal microscopic imaging with X-Y projections (Figure 1B). For microscopic control of phagocytosis, cells were 5-fold diluted in ice-cold PBS and sedimented onto a coverslip for 30 min. Later cells were fixed with 4% (w/v) paraformaldehyde and analyzed with a Zeiss LSM710 confocal laser scanning microscope equipped with 40×/1.3 and 63×/1.3 oil immersion objective (Plan-Neofluar, Zeiss). Images were analyzed with LSM Image Browser software (Zeiss).

### Activation of Adherent Neutrophils

Selective activation of Mac-1 complex of adherent human neutrophils was performed in 6 well tissue culture plates (Biofil, Hungary) coated overnight with 0.2 mg/mL BSA or 50 μg/mL C3bi (both from Merck, Darmstadt, Germany) as previously described (33). To obtain immobilized immune complex–coated surfaces, human lactoferrin (20 μg/mL; Sigma-Aldrich, USA) was covalently linked to poly-l-lysine (Sigma-Aldrich, USA) coated 6 well plates and then treated with polyclonal anti-lactoferrin (LTF) IgG (1:400 dilution; Sigma-Aldrich, USA) or non-specific IgG (1:400 dilution; Sigma-Aldrich, USA) for 1 h as previously described (34). Unbound immunoglobulin was removed by washing the plate by HBSS three times. The isotype control serves to test the unspecific binding of applied antibodies.

### Bacterial Survival Assay

Opsonized bacteria (5 × 10^7^/50 μL HBSS) were added to 500 μL EV (derived from 5 × 10^6^ PMN) suspended in HBSS. During a 40 min co-incubation step at 37°C the bacterial count decreases or increases depending on the samples' antibacterial effect and the growth of bacteria. At the end of the incubation, 2 mL ice-cold stopping solution (1 mg/mL saponin in HBSS) was added to stop the incubation and lyse EVs. After a freezing step at −80°C for 20 min, samples were thawed to room temperature and inoculated into LB broth. Bacterial growth was followed as changes in OD using a shaking microplate reader (Labsystems iEMS Reader MF, Thermo Scientific) for 8 h, at 37°C, at 650 nm. After the end of growth phase the initial bacterial counts were calculated indirectly using an equation similar to PCR calculation, as described previously (35).

### Statistics

Comparisons between two groups were analyzed by two-tailed Student's *t*-tests or ANOVA. Exact statistical tests are indicated in the figure legends. All bar graphs show mean and ± SEM. Difference was taken significant if *P* value was < 0.05. ^*^ represents *P* < 0.05; ^**^ represents *P* < 0.01; ^***^ represents *P* < 0.001. Statistical analysis was performed using GraphPad Prism 6 for Windows (La Jolla, CA, USA).

## Results

### Comparison of Receptors Involved in Phagocytosis and EV Generation Initiated by Opsonized Particles

We first carried out a detailed analysis on the involvement of different receptors in phagocytosis, using differently opsonized particles (Figure 1). Following the process by flow cytometry up to 30 min, we could detect only minimal phagocytosis of non-opsonized bacteria by human neutrophils (Figure 1A). If bacteria were treated with complement-depleted serum and so opsonized mainly by antibodies that activate different Ig-binding FcR, we observed phagocytosis in ~30% of the cells (Figure 1A). In contrast, particles opsonized in full serum, allowing thus the activation of both Fc and complement receptors, induced significantly greater phagocytosis, and bacteria were detectable in ~80% of the investigated neutrophils (Figure 1A). In Figure 1B we show the results of similar experiments carried out by confocal microscopy, verifying that bacteria were in fact engulfed, not only associated to the surface of the cells. The kinetic experiments presented in Figure 1A indicated that under our conditions phagocytosis was completed in 20 min. Therefore, in the following experiments only data obtained by flow cytometry at 20 min are shown.

In order to confirm the complement receptor playing major role in phagocytosis under our conditions, we tested neutrophils from genetically modified mice. Earlier studies (1) suggested CR3-which is identical with the β_2_ integrin Mac-1—as the most important complement receptor in neutrophilic granulocytes. In accordance with previous findings (3), deletion of either CD18, the common β chain of all neutrophil β_2_ integrins, or CD11b, the specific α chain of Mac-1 resulted in drastic decrease of phagocytosis (Figures 1C,D). In contrast, depletion of CD11a, the α chain of the integrin LFA-1, which does not serve as complement receptor, had no influence on phagocytosis (Figure 1E).

**Figure 2:**
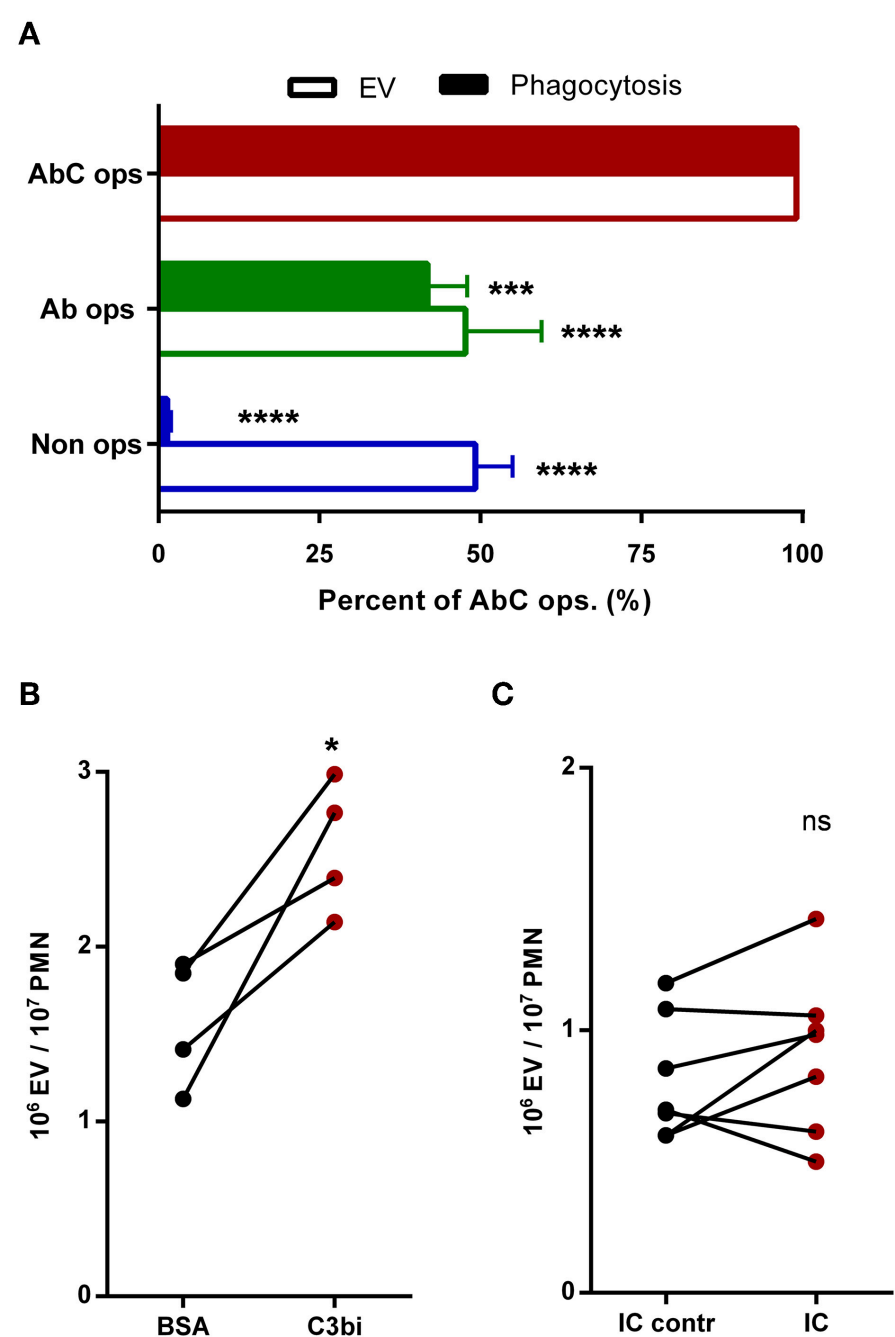
Participation of different receptors in activated EV generation from human neutrophils. **(A)** Comparison of phagocytosis and EV production after 20 min activation by different opsonized particles. +SEM, *n* = 21 (EV) or 8 (phagocytosis). Data were compared using RM two-way ANOVA coupled with Dunett's *post hoc* test. **(B)** Generation of EV by neutrophils adherent to non-specific BSA or specific C3bi surface. **(C)** Generation of EV by neutrophils adherent to immune complex surface. Data were compared using paired Student's *t*-test; *n* = 4 and 7. *P < 0.05; ***P < 0.001; ****P < 0.0001.

Next we investigated EV production from human neutrophils under similar conditions. As shown in Figure 2A, non-opsonized particles induced moderate EV generation, which was not further increased if particles were opsonized by antibodies. Fully opsonized particles, which were able to stimulate both Fc and CR receptors, initiated maximal EV release. To substantiate the difference in the role of Fc and CR receptors in EV biogenesis, in the following experiments we stimulated the two types of receptors selectively on coated surfaces (Figures 2B,C). C3bi, the specific ligand of CR3 receptor induced significant and consistent increase of EV production from adherent neutrophils (Figure 2B), whereas no significant change of EV release was observed if the cells were seeded on an immune complex surface (Figure 2C). Thus, under our experimental conditions Mac-1/CR3 seems to play a decisive role both in phagocytosis and in EV generation. In the following experiments we focused on the signaling process downstream of Mac-1.

### Role of the Adaptor Proteins FcRγ and DAP12 in Mac-1 Initiated EV Generation and Phagocytosis

Outside-in signaling of β_2_ integrins was shown to involve tyrosine kinases, but the short intracellular segments of both chains lack suitable signaling sequences. The adapter molecules FcRγ chain and DAP12 which contain conserved immune receptor tyrosine based activation motives (ITAMs) were shown to play a critical role in downstream signaling of β_2_ integrins (36). Therefore, we investigated first the involvement of these two molecules in EV generation and phagocytosis in neutrophils obtained from genetically deficient mice. EV production was detected both under resting conditions (spontaneous, sEV) and upon stimulation with full murine serum opsonized zymosan particles (activated, aEV).

**Figure 3:**
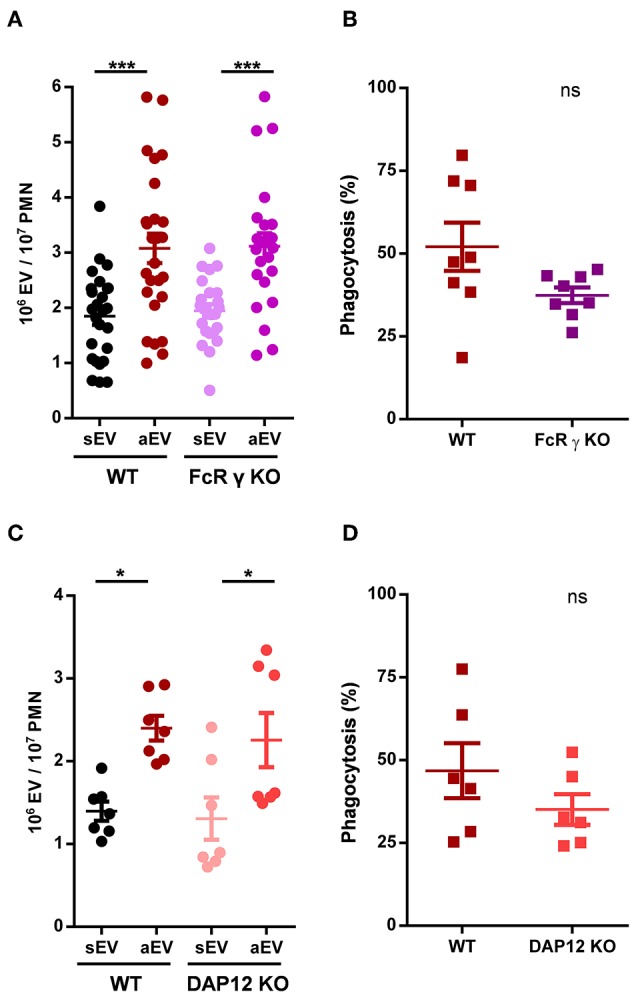
Role of adaptor proteins in aEV generation and phagocytosis of murine neutrophils. Comparison of sEV and aEV production of WT vs. FcRγ chain KO **(A)** and DAP12 KO **(C)**, murine PMN. Comparison of phagocytosis of fully opsonized bacteria by WT vs. FcRγ chain KO **(B)** and DAP12 KO **(D)** murine PMN. Data were compared using Student's *t*-test; *n* = 24, 8, 7, 6 +SEM. *P < 0.05; ***P < 0.001.

Deletion of FcR γ chain prevents signal transduction via all FcR in murine neutrophils (34) and it was shown to transmit Mac-1 signaling as well (36). In FcRγ KO animals we did not see any change in EV production as compared to the wild type, and observed only a minor, statistically non-significant decrease of phagocytosis (Figures 3A,B). The latter finding is in accord with the moderate activity of FcRs shown in Figure 1. Similar results were obtained in neutrophils from DAP-12 deficient animals (Figures 3C,D) indicating that neither adapter molecule plays an exclusive role in Mac-1 signaling, and suggesting a potential redundant function.

### Src-Family Kinases and Syk Are Dispensable for EV Generation but Required for Phagocytosis

Phosphorylation of the ITAM sequences of the adapter molecules is carried out by the Src family kinases (SFK), of which Hck, Fgr, and Lyn are expressed in murine neutrophils (37). We investigated aEV production and phagocytosis in neutrophils from triple KO mice, which lack all three SFKs.

**Figure 4:**
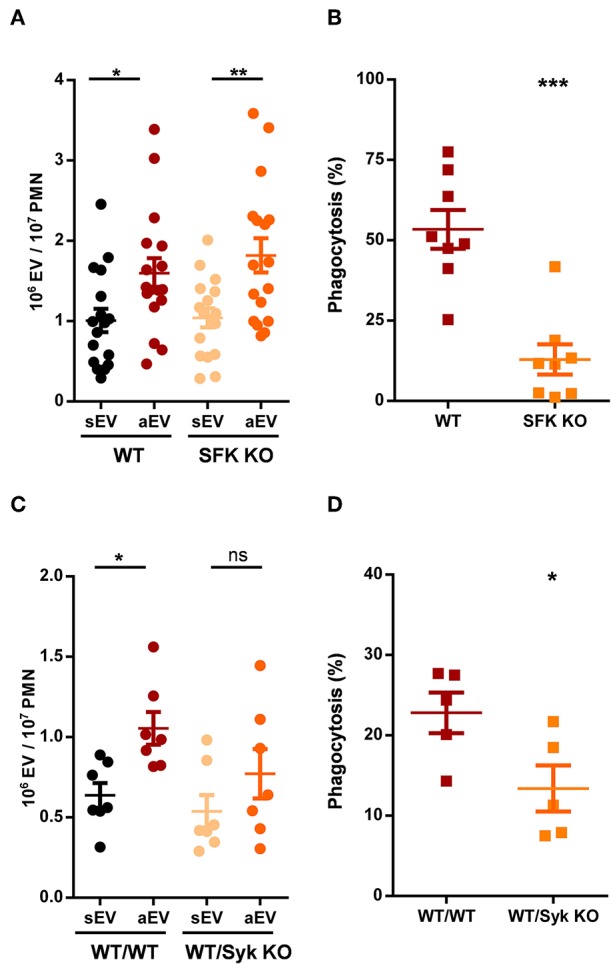
Role of tyrosine kinases in aEV generation and phagocytosis of murine neutrophils. Comparison of sEV and aEV production of WT vs. SFK KO **(A)** and Syk KO **(C)** murine PMN. Comparison of phagocytosis of fully opsonized bacteria by WT vs. SFK KO **(B)** and Syk KO **(D)** murine PMN. Data were compared using Student's *t*-test; *n* = 17, 8, 7, 5 +SEM. *P < 0.05; **P < 0.01; ***P < 0.001.

Phagocytosis of fully opsonized bacteria was significantly impaired in the absence of all three SFKs. Surprisingly, EV generation was not affected at all and reached similar values as detected with wild-type cells (Figures 4A,B). Thus, at this point we observed serious divergence in the signalization process of phagocytosis and aEV generation.

Phosphorylation of the ITAM sequences of the adaptor proteins by SFKs allows the binding and activation of Syk tyrosine kinase that plays important role in the transmission of several SFK mediated cell functions (4). Syk-deficient animals are not viable but the function of the hematopoietic compartment can be studied following transplantation with wild-type or Syk-deficient bone marrow. The data shown in Figures 4C,D were obtained using neutrophils of transplanted animals. Generation of aEVs from Syk-deficient neutrophils was not significantly different from the extent obtained with neutrophils following transplantation of wild-type bone marrow, although the increase compared to sEV generation proved not to be statistically significant either (Figure 4C). Phagocytosis by neutrophils from transplanted animals was very low, nevertheless significant difference was obtained between neutrophils produced after transplantation with wild-type or Syk KO bone marrow (Figure 4D). Thus, neither SFKs nor Syk seem to be essential for the production of aEVs in neutrophilic granulocytes whereas they are required for maximal phagocytosis.

### Requirement for Calcium Signaling Is Different in EV Generation and Phagocytosis

**Figure 5:**
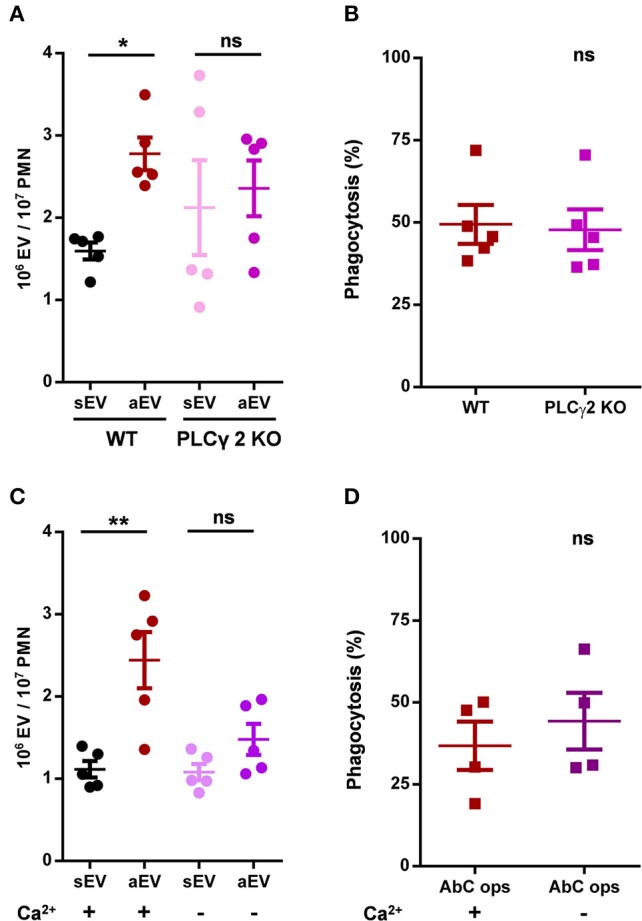
Role of PLCγ2 and availability of extracellular calcium in aEV generation and phagocytosis of murine neutrophils. Comparison of sEV and aEV production of **(A)** and phagocytosis of fully opsonized bacteria by **(B)** WT vs. PLCγ2 KO murine PMN. Quantification of aEV production from **(C)** and phagocytosis by **(D)** WT murine PMN in the presence and absence of extracellular Ca^2+^. Data were compared using Student's *t*-test; *n* = 5, 5, 5, 4 +SEM. *P < 0.05 and **P < 0.01.

The γ2 isoform of phospholipase C was shown to be essential in organization of several neutrophil functions initiated by β_2_ integrin stimulation (23). Hence we investigated aEV production and phagocytosis in neutrophils of PLCγ2 deficient animals. Phagocytosis was not influenced, but aEV production was seriously impaired (Figures 5A,B). In fact, there was only very little difference between spontaneous and activated EV generation from PLCγ2 KO neutrophils (Figure 5A). Thus, the key enzyme of inositol trisphosphate (IP3) generation and consequent calcium signaling seems to be required for aEV generation but it is dispensable for phagocytosis.

Next, we investigated whether presence of calcium in the extracellular space is essential for aEV generation or phagocytosis. As shown in Figures 5C,D for murine neutrophils, aEV production was almost completely inhibited in the absence of calcium, whereas phagocytosis was not affected at all. Interestingly, neither the absence of calcium, nor the lack of PLC γ2 decreased spontaneous EV release.

**Figure 6:**
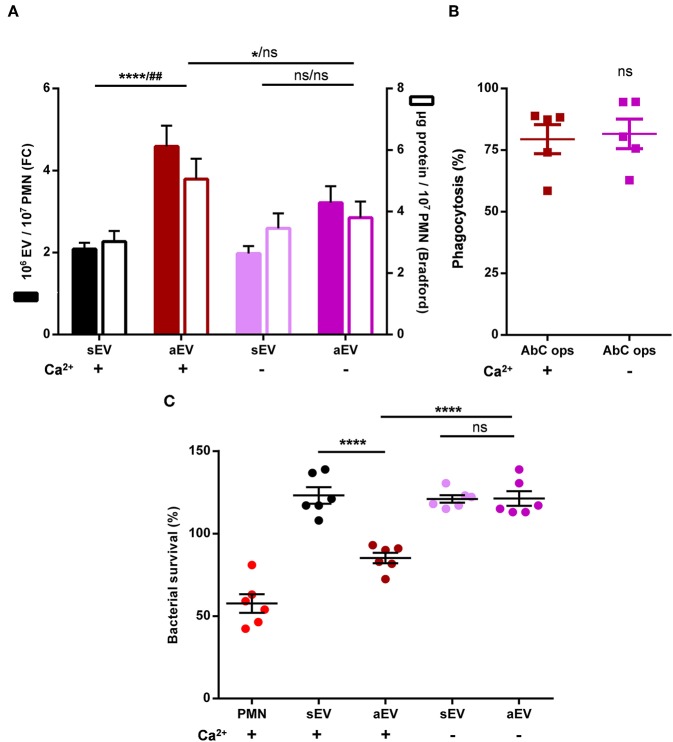
Role of extracellular calcium in aEV production and phagocytosis of human neutrophils. Quantification of aEV production from **(A)** and phagocytosis by **(B)** human PMN in the presence and absence of extracellular Ca^2+^. Production of aEV was assessed on the basis of detectable EV number (left axis) and total protein content (right axis); aEV production data were compared using RM two-way ANOVA coupled with Tukey's *post hoc* test; *n* = 11. Level of significance is indicated by * for EV numbers and by # for protein amount. Phagocytosis was analyzed using Student's *t*-test; *n* = 5. **(C)** Effect of aEV produced in the presence or absence of extracellular Ca^2+^ on bacterial growth. Data were compared using RM-ANOVA coupled with Tukey's *post hoc* test; *n* = 6.

In the following experiments, we wanted to substantiate our observations also in human neutrophils, where the antibacterial function could be tested as well. EV generation was followed on the basis of two parameters: number of vesicles detectable by flow cytometry and total protein content of the sedimented vesicles. Both parameters indicated defective aEV production in the absence of extracellular calcium whereas sEV release was not affected (Figure 6A). In contrast, there was no difference in phagocytosis in the presence or absence of external calcium (Figure 6B). Thus, human PMN behaved similar to their murine counterparts.

Antibacterial effect of the separated vesicles was followed under conditions where the effect of EVs of identical protein content was tested upon bacterial survival. Intact human neutrophils were applied as positive control. As indicated in Figure 6C, only EVs produced upon stimulation with opsonized particles in the presence of external calcium were able to impair bacterial growth whereas EVs produced upon the same stimulation but in the absence of calcium lacked the antibacterial effect.

## Discussion

In this study we investigated two cellular processes initiated by opsonized particles and show that both in generation of EVs with antibacterial property and in phagocytosis the multifunctional surface molecule Mac-1 plays the central role. However, our data indicate that aEV production is independent of phagocytosis in spite of being triggered by the same receptor. On one hand aEV generation proceeds also on coated surface where phagocytosis is not possible (Figure 2B) and on the other hand we observed major differences in the implicated signaling pathway (Figures 4–6). Our findings suggest that the cytoskeletal rearrangement related to phagocytosis is neither sufficient nor required for EV generation in neutrophils.

**Figure 7:**
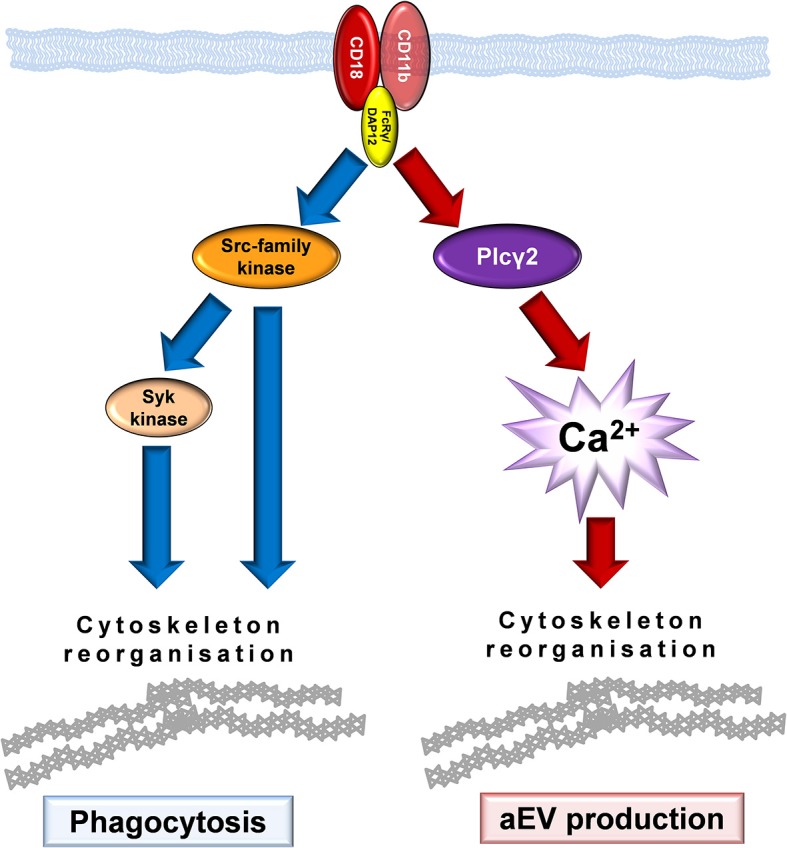
Model of Mac-1 signaling initiating phagocytosis or production of antibacterial EVs.

Mac-1 is one of the two major β_2_ integrins which plays an important role in slow rolling and adherence of neutrophilic granulocytes (2, 3, 38). β_2_ integrin-dependent adherent activation of neutrophils which consists of spreading, superoxide production and degranulation was shown to involve activation of SFKs, Syk tyrosine kinase and PLCγ2 enzymes (23, 25, 39–41). Specific Mac-1 dependent degranulation was shown to depend on SFK and Syk kinase activity (42). Our current data indicate that the two investigated Mac-1 dependent processes are only partially dependent on this previously characterized signaling pathway. Phagocytosis entails the SFK and Syk tyrosine kinases, but it proceeds undisturbed in the absence of PLCγ2 and extracellular calcium (Figure 7). Much to our surprise, enhanced EV production triggered by Mac-1 did not depend on activity of SFK and was not affected by their absence. In contrast, activated EV production was seriously compromised by the lack of PLCγ2, the enzyme producing the calcium mobilizing messenger molecule IP3. Similarly, no significant increase of EV production could be achieved by Mac-1 stimulation in the absence of extracellular calcium either in murine or in human neutrophils. Last, but not least EVs produced upon encountering opsonized particles in the absence of extracellular calcium, had no antibacterial effect. The fact that phagocytosis was not affected by the absence of extracellular calcium indicates that receptor binding was functioning properly. Apparently, generation of antibacterial EVs requires concurrent calcium entry and intracellular calcium signaling. In different cell types Ca ionophores were shown to initiate EV generation (43, 44), although the functional properties of those vesicles were not investigated in details. In contrast, here we demonstrate - to our knowledge the first time - the key role of calcium signaling in biogenesis and function of EVs triggered by physiological stimuli via an identified receptor.

Freshly isolated resting neutrophils produce a basal amount of EVs spontaneously (sEV) which served as reference in all our experiments. Interestingly, this constitutive EV production did not depend on any signaling element investigated in our study. This fact clearly indicates the existence of distinguishable signaling pathways in production of EVs of different properties and functions.

Mac-1 is a multifunctional molecule with over 40 identified ligands (45). The ligands investigated in detail were shown to bind to partially overlapping but distinct sites and some were suggested to behave as biased agonist (46). Our studies extend this picture with a Mac-1 dependent pathway that proceeds without participation of SFK (and probably Syk) kinases. Previous studies indicated that both SFK, Syk and PLCγ2 seem to be dispensable for β_2_ integrin dependent migration (19, 23, 25, 47), although the involvement of Mac-1 remained questionable. In another study (42) Mac-1 dependent elastase release was only partially inhibited in the absence of all 3 SFK. All these observations suggest the possibility of multiple parallel signaling pathways in organization of different cellular responses triggered by the same cell surface receptor. Thereby our findings raise the potential of selective modulation of aEV production without interference with the phagocytic process.

## Data Availability Statement

All datasets generated for this study are included in the article/supplementary material.

## Ethics Statement

The studies involving human participants were reviewed and approved by Budapest Capital Government Office, Public Health Direction. The patients/participants provided their written informed consent to participate in this study. The animal study was reviewed and approved by Animal Care Committee of the National Authority for Animal Health (Budapest, Hungary).

## Author Contributions

ÁL and EL designed the experiments. ÁL, VS, BB, and DS carried out the experiments. AM provided expertise with genetically modified animals. ÁL and EL summarized the results and wrote the manuscript. EL obtained the funding.

### Conflict of Interest

The authors declare that the research was conducted in the absence of any commercial or financial relationships that could be construed as a potential conflict of interest.
